# No evidence for punishment in communally nursing female house mice (*Mus musculus domesticus*)

**DOI:** 10.1371/journal.pone.0179683

**Published:** 2017-06-22

**Authors:** Manuela Ferrari, Barbara König

**Affiliations:** Department of Evolutionary Biology and Environmental Studies, University of Zurich, Zurich, Switzerland; Istituto Superiore Di Sanita, ITALY

## Abstract

Punishment is claimed as an important mechanism to stabilise costly cooperation in humans, but its importance in social animals has been questioned recently due to both conceptual considerations and a lack of empirical evidence (only few published studies). We empirically tested whether there is evidence for punishment in communally nursing house mice (*Mus musculus domesticus*, direct descendants of “wild” animals). Communally breeding females pool their litters and raise all offspring together, indiscriminately caring for own and other offspring. Such a situation resembles a public good and provides scope for exploitation if females vary in their relative contributions to the joint nest (offspring number). We allowed two females to communally breed and conducted removal experiments both in the presence and absence of pups. We aimed to test whether reduced investment by one of the females (induced through separation from the partner and their combined offspring for 4 or 12 hours) leads to increased aggression by the other female after the reunion. We found no evidence for punishment, on the contrary, females increased socio-positive behaviours. The costs of losing a partner in a communally breeding species might be too high and hinder the evolution of punishment. Our findings add to a growing list of examples questioning the role of punishment in cooperating non-human animals and emphasise the importance of empirical testing of its assumptions and predictions.

## Introduction

Cooperative offspring care is a wide spread phenomenon in different taxa [1, 2] and refers to the situation in which individuals help to raise offspring that is not their own. Reproductive skew varies among caring individuals, ranging from despotic systems with one breeding pair and several non-reproducing helpers (cooperative breeding), to egalitarian groups with several breeding females raising their offspring together in one nest (communal breeding) [3–5].

Caring for another female’s offspring and especially nursing non-offspring requires an evolutionary explanation, considering the high energetic costs of parental care in general and in particular of lactation in mammals [6]. Cooperation in these situations is expected to be evolutionary stable only when individuals gain indirect or direct fitness benefits [7–9].

Communal breeding or nursing seems at first free of conflict because all females involved gain direct fitness benefits (in the form of reproduction), even more so in species in which females preferentially nurse their own young [10]. However, in many species females indiscriminately nurse all young in the nest and are unable to discriminate between own and other offspring [11–15]. Different contributions to the joint nest in terms of offspring numbers will then result in different benefits for the females involved. During indiscriminate communal nursing individuals can increase their benefit by lowering investment at the cost of their partners, creating a social dilemma [16] that resembles a public good problem (i.e. tragedy of the commons [17, 18]). Females that reduce their investment or contribute more offspring to the joint nest than their partners will exploit the public good (i.e. the indiscriminate cumulative care provided to all young) [15].

Adjusting parental care to the expected fitness benefits (i.e. the expected contribution to the nest) would be a beneficial strategy to avoid overinvestment and can be observed in birds and fish, with males adjusting their overall investment to the rate of extra pair paternity [19–21]. They reduce feeding behaviour when the proportion of their own offspring in the nest is smaller. In a similar way, in species that cannot discriminate own from other offspring, females could reduce their overall investment when their contribution to the joint brood or litter is smaller than their partner’s. Alternatively, females could decrease their investment to gain an advantage, which will however aggravate the conflict. So far there is no evidence that lactating females adjust their investment to their own litter size in species with indiscriminate care, but rather that they invest according to the total number of offspring in the nest [14, 15, 22], raising the question what prevents females from reducing their investment?

Aggression or punishment by the other females might be one way to prevent a female from lowering her investment, enforcing continued cooperation. Punishment has been discussed as a mechanism to suppress individual selfishness in a social dilemma, and proved to stabilise cooperation in humans [23–25]. There are, however, hardly any examples known in animal systems. Leighton and Meiden [26] showed that sociable weaver increased their investment into the public good (nest building) after having been attacked, and the threat of evictions seemed to serve as a stabilising mechanism in communally breeding banded mungooses [27].

In cooperative breeders, which are characterised by a strict dominance hierarchy with only the dominant female or pair reproducing, the role of punishment has been studied in a variety of species. The pay-to-stay hypothesis developed for cooperative breeders assumes that subordinate helpers have to pay rent in the currency of investment into the offspring to be allowed on the territory, or as a member of the group [28, 29]. In birds [30], fish [29] and invertebrates [31], it has been shown that the dominant pair (or the dominant male alone) punishes “lazy” helpers, therefore stabilising cooperation. Raihani *et al.* [32] challenged those findings because of lacking evidence that punishment changes the behaviour of the target individual, which is necessary for such a mechanism to work. However, Fischer *et al.* [33] argue in their recent paper that they found evidence in a cichlid species (*Neolamprologus pulcher*) that punishment indeed increased helping behaviour in small groups, where the dominant pair can monitor individual helping behaviour of subordinates.

Given the controversial discussion of punishment as a stabilising factor for cooperation outside of humans and due to the lack of studies analysing its importance in public good situations in non-human animals, we empirically tested the situation of a social dilemma in a communally breeding mammal. House mice are a good study species to analyse whether there is evidence for coercion in a communal breeder. Female house mice show two breeding strategies, rearing their young either solitarily or together with one or several other females in a communal nest [2, 34–36]. During communal nursing females invest milk according to the total number of offspring in the nest and not their own litter size [15], resulting in females benefiting unequally if they differ in litter size. Females seem unable to discriminate between own and other young, making it impossible for them to selectively nurse only own young [13].

Since conflict is expected to be highest among unrelated individuals, we used previously unfamiliar and unrelated females (descendants of wild house mice) in a laboratory experiment and allowed them to form a communal nest. One female was then removed from the communal nest for 4 and—on a separate day—for 12 hours, once while females were rearing young and once as a control while they had no offspring. After 4 hours without suckling stimulus by pups milk production decreases [37]. Nevertheless since in a wild population females may not visit their litters for even longer periods of time (communally reared pups in a wild population were found on average to be left alone 11.7 hours per day [36]), we also chose a second separation period of 12 hours to increase the conflict potential.

This experiment allowed us i) to assess how females reacted after the “lazy” partner returned and ii) to quantify whether the remaining female compensated for the absence of her partner by increasing the amount of time she spent nursing. If punishment or coercion play a role in stabilising cooperation among communally nursing females, we expected increased aggression after the removed female is returned only in the presence, but not the absence of pups.

## Materials and methods

### Animals and husbandry

We used F1 to F3 descendants of wild caught house mice as study animals. The population of origin lived near Zurich (Switzerland), see [38] for more information. Mice were kept in the laboratory under a standardised light:dark cycle (14:10h, light on at 05:30 hours CET and constant temperature of 22-24°C). Experimental mice originated from monogamously kept breeding pairs and stayed in the parental cage until weaning (day 23). Afterwards they were kept in Macrolon Type III cages (23.5x39x15cm) together with same-sex siblings until the beginning of the experiment. Food (laboratory animal diet for mice and rats, no. 3430, Kliba) and water were provided *ad libitum*. Cages of all mice were enriched with papertowels and cardboard, serving both as cover and nest building material.

### Experimental design

**Fig 1:**
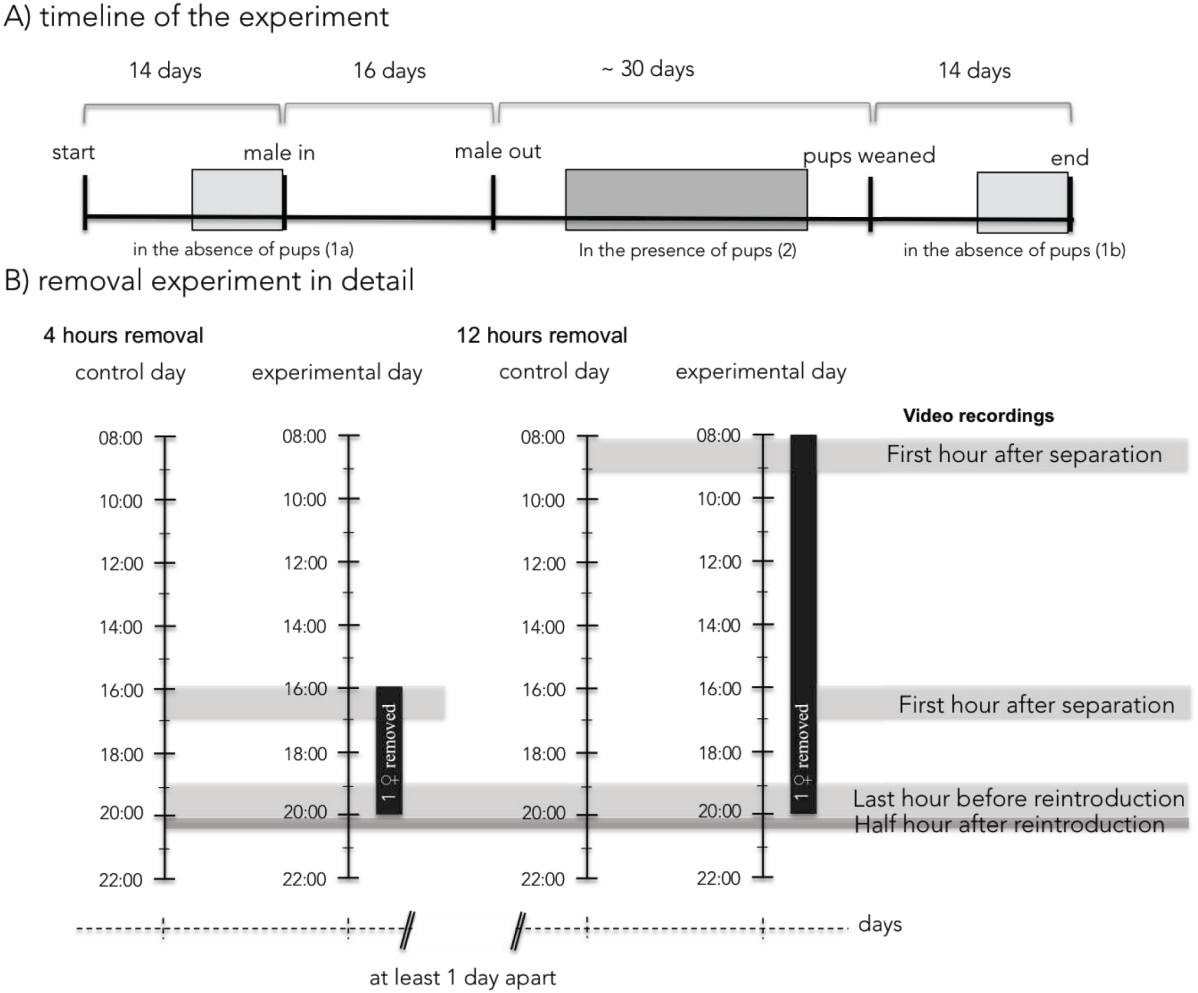
A timeline of the experiment. A) Highlighted are the periods during which the removal experiments were conducted in the absence of pups (for half of the trials in period 1a, for the other half in period 1b) and in the presence of pups (period 2). B) Detailed plan of the actual removal experiments and the corresponding video recordings used for behavioural analyses. The videos before the reintroduction of the “removed” female (first hour after separation and last hour before reintroduction) were only analysed for the “resident” females and only in the presence of pups.

25 pairs of genetically unrelated and unfamiliar (reared apart) female house mice, not more than 12 days apart in age (mean age difference: 4.3 days), were kept together in a cage system comprised of three Macrolon Type II cages (18x24x14cm), connected via transparent plastic tubes. Females were sexually mature but naive at the beginning of the experiment, ranging in age from 44 to 117 days (mean ± SE: 69.2 ± 4.5 days). After two weeks an unrelated and unfamiliar male (mean ± SE: 96 ± 9.5 days) was introduced to each pair of females. On day 16 after the introduction, the male was removed again from the cage system (see Fig 1A for a detailed timeline). We checked the cages daily for new litters and documented the occurrence of the first communal nest (two litters born in the same nest within maximally 16 days; [5]). Female weight and body condition were assessed at the onset of the experiment, at the introduction of the male, and at the end of the experiment.

#### Female body condition

Females were regularly checked for signs of aggression. According to Swiss animal law, animals were separated in the case of aggression that did or might result in severe injuries, indicated by the occurrence of bleeding open wounds on the animal’s back or tail. However, house mice also frequently bite each other in the tail during aggressive interactions without serious consequences. Such harmless bites result in small scars visible on the tails. We categorised the occurrence of such scars the following way: 0) no scars on the tail, 1) one or very few scars visible, 2) several scars or tip of the tail missing, and 3) many scars and/or fresh bites on back and tail. As described before, mice falling into the third category were immediately separated from their partner and the experiment stopped. The occurrence of small scars or wounds was documented at the beginning of the experiment, immediately before the introduction of the male and at the end of the experiment.

#### Removal

One of the two females within a pair was randomly assigned to the category “removed”, the other female was categorised as “resident” female. During a removal the female of the “removed” category was separated by enclosing her in one of the three cages, with the help of a shutter in the tube connecting the cages. Both females therefore remained in their home cage system and still had olfactory, auditory and partially even visual contact with each other. Such procedure is expected to minimise the stress for the separated animal. The “resident” female remained in the larger part of the cage system (two cages), and the nest was always located within her part. Females produced nests with the provided paper towels also while they were not breeding.

Two removal experiments were conducted over the duration of the experiment; once in the absence of pups (time period 1a or 1b, see Fig 1A) and once in the presence of pups (time period 2, see Fig 1A). For half of the pairs the removal in the absence of pups took place before the introduction of the male (during the second week after the onset of the experiment) and in the other half after all offspring were weaned and removed from the cage (when 23 days old). One removal experiment comprised two separate removal events, first over 4 and then over 12 hours, with at least two days separating them. Fig 1B describes the removal in more detail.

#### Behavioural analyses

Videos for behavioural analyses were recorded during the removal (60min each) and after the reintroduction of the “removed” female (30min, see Fig 1B). Recordings were conducted on the day of the removal and during the same time periods on the day before as a control. The change in the behaviour in comparison to the control day [experimental day—control day] was used for further analysis. By analysing the difference and not the absolute values, we minimised confounding effects by other factors, such as the number of pups females had or the age of the pups during the removal.

Table 1 summarises the behaviours documented during the periods of observation in the absence and in the presence of pups.

**Table 1:** Behaviours recorded in the observation sessions (after reintroduction and during the removal) in the presence and absence of pups.

behaviour	description
**After the reintroduction of the “removed” female (in the presence and absence of pups)**	
Socio-positive behaviours	
time spent resting with body contact	total time two females spent resting with bodycontact [s]
allogrooming	number of bouts within 30min a female allogroomed the other one
Socio-negative behaviours	
biting	number of times a female bit the other
chasing	number of times a female chased the other
Neutral behaviours	
sniffing nose	number of times a female sniffed the other mouse’s nose
sniffing anogenital region	number of times a female sniffed the other mouse’s anogenital region
nursing (only in the presence of pups)	total time a female spent on the young in the nest [s]
**During the removal (only in the presence of pups)**	
nursing	total time the “resident” female spent on the young [s]

### Statistical analyses

All statistical analyses were performed with R 3.02 [39]. Linear (or generalised linear) models ((G)LM) and Linear mixed models (LMM) were used for analysis. Mixed models were performed with the package lme4 [40]. Fulfillment of model assumptions was tested visually (qqplots were used to assess whether residuals were normally distributed and standardised residuals were plotted against fitted residuals to check for homogeneity of the variance) and parametric bootstrapping was used to assess the significance of fixed effects. The package psych [41] was used to perform a maximum likelihood factor analysis.

The behaviours recorded from both females after the reintroduction of the “removed” mouse were first analysed with a maximum likelihood factor analysis, following the methodology described in [42]. In a first step, the correlation matrix between the six behavioural traits observed (S2 Table) was tested for suitability to conduct a factor analysis. Both the Bartlett’s test of sphericity (*χ*^2^ = 439.61, df = 15, p < 0.0001) and the Kaiser-Meyer-Olkin factor adequacy test (KMO = 0.59) indicated the data’s suitability to continue with the factor analysis.

Visual inspection of the scree plots and a parallel analysis after Horn [42, 43] suggested to conduct the factor analysis with two factors. The matrix of loadings was rotated (varimax rotation) to obtain orthogonal factors. A detailed table with the loadings on both factors can be found in S1 Table. The behaviours chasing and biting loaded heavily on factor 1 (0.99 each), together with a moderate loading of sniffing at another mouse’s anogenital region (0.46). We consequently interpreted factor 1 as socio-negative behaviours and the scores on factor 1 were used for further analyses. In contrast, sniffing at another mouse’s nose (0.71) and allogrooming (0.33) loaded on factor 2. We interpreted factor 2 as socio-positive or, more generally as non-aggressive interactions and used the scores for further analyses. The loadings of the clearly positive behaviours allogrooming (0.33) and resting with body contact (-0.25) were not particularly high, while there was a high loading of sniffing at another mouse’s nose. It is difficult to classify sniffing as either negative or positive, though in a “greeting” context we interpreted it as positive if not followed by aggression. Sniffing at another mouse’s anogenital region loaded both on factor 1 (0.46) and factor 2 (0.63), indicating for it to be a more neutral behaviour in relation to the quality of the relationship between two females and to occur in negative as well as neutral or socio-positive situations.

### Ethical note

The experiment has been approved by the Veterinary Office Zurich, Switzerland (licence no. 90/2014).

## Results

### Compatibility of female pairs

Twelve out of 25 pairs of females had to be separated before the first female gave birth, because of aggression between the females. Eight pairs were separated within the first five days of the experiment and four were separated after the introduction of the male. In 11 of the remaining 13 pairs both females gave birth and formed a communal nest. In one pair each, females failed to conceive, or one female died while giving birth. The first communal nest on average was formed after 63.9± 8.8 days (mean ± SE), with a total litter size ranging from 7 to 14 pups (mean ± SE: 12± 0.7 pups). The average age difference between the two litters in a communal nest was 2.5± 0.6 days (mean ± SE).

Neither the initial difference in weight nor in age between the two females had a significant effect on a pair’s probability to be compatible (meaning that females did not have to be separated due to aggression) (GLM, weight difference: F_1,22_ = 33.02, p = 0.79, age difference: F_1,21_ = 29.53, p = 0.10).

### Female body condition

There was no significant difference between “removed” and “resident” females in the number of scars on their tails at the end of the experiment (Wilcoxon signed-rank test, *N* = 22, W = 65, p = 0.76). Overall females had more scars at the end of the experiment compared to before the introduction of the male (LM, F_1,40_ = 9.25, p = 0.004). This was, however, independent of whether a female was the “removed”or the “resident” female (LM, F_1,40_ = 0.18, p = 0.67), indicating that the removal did not result in increased aggression towards the “removed” female.

### Socio-negative and socio-positive behaviours after reintroduction of the removed female

**Fig 2:**
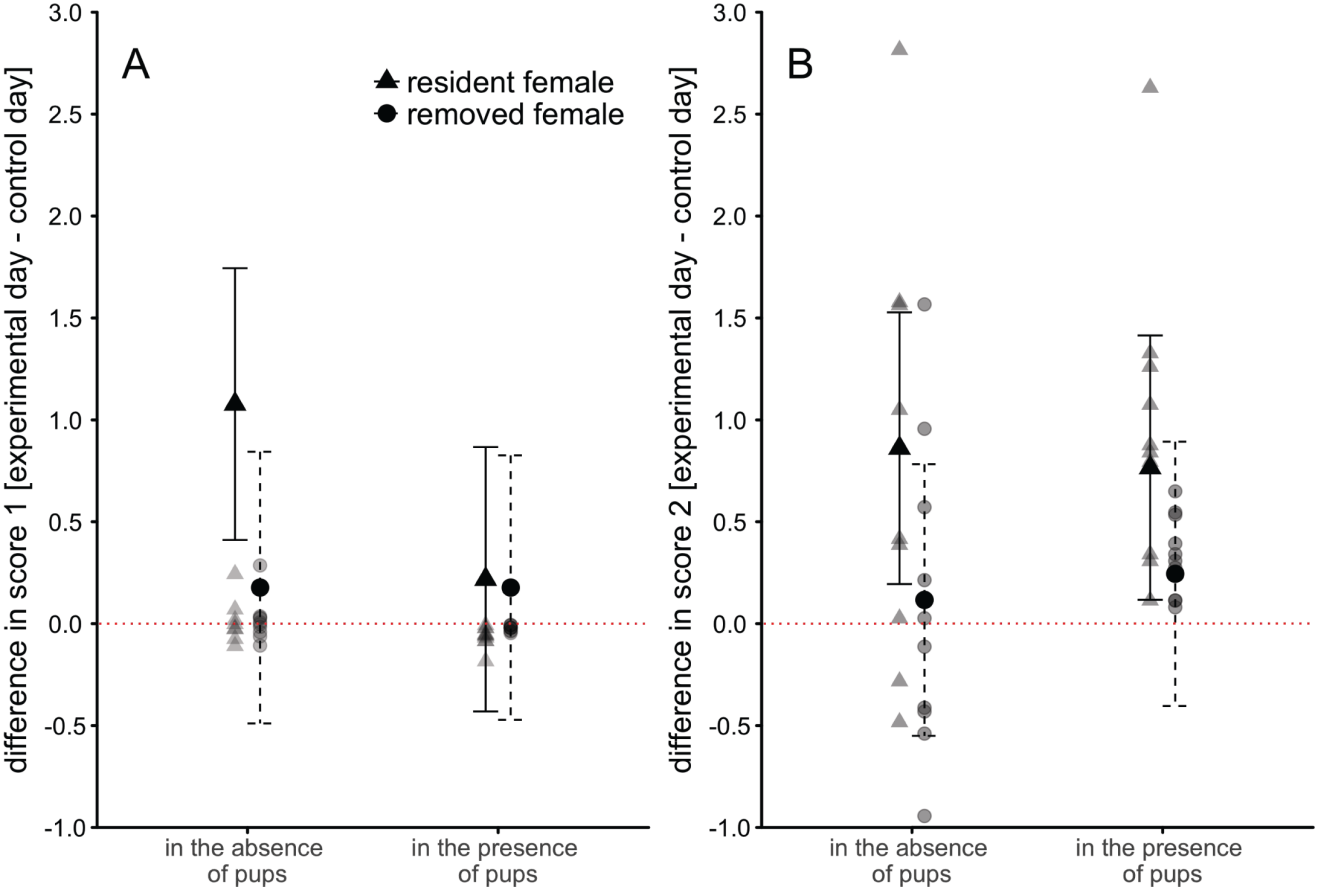
Socio-negative and socio-positive behaviours shown after the reintroduction. Change in the occurrence of A) socio-negative and B) socio-positive behaviours (factors 1 and 2 from a maximum likelihood factor analysis) shown by communally nursing female house mice after one female had been removed prior to the observation compared to control observations the day before (without a removal). Displayed are model means and the 95% CI of the mean for the 4 hours removal only, because there was no significant difference between the 4 and 12 hours removals. Raw data are plotted in grey. Two outliers for the “resident” female in the absence of pups are omitted from the figure (they would be at 6.5 and 11.3; the values were nevertheless included in the statistical analyses).

We found no evidence that the removal and later reintroduction of a female significantly influenced the occurrence of socio-negative behaviours in the presence of pups. Only the “resident” female in the absence of pups showed increased socio-negative behaviour that was significantly different from 0 (confidence interval of the mean did not cross 0, see Fig 2A). There was, however, no significant difference between the “removed” and the “resident” female (*χ*^2^_1_ = 4.48, p = 0.133, see Table 2a) in socio-negative behaviour, nor was there a significant interaction between whether a female was “removed” or not and the presence or absence of pups (LMM, *χ*^2^_1_ = 2.25, p = 0.166, see Table 2a).

**Table 2:** Summary statistics for the linear mixed models. Given are estimates and SE for all explanatory variables contained in the models analysing (a) socio-negative and (b) socio-positive behaviours. The intercept in both models corresponds to “resident” female in the absence of pups after the 4 hour removal.

	explanatory variable	estimate	SE	*χ*^2^	p
a) **response variable:** change in socio-negative behaviours (factor 1) compared to the control day					
	intercept	1.08	0.33	–	–
	in the presence of pups	−0.86	0.40	4.40	0.13
	removed or resident female	0.90	0.41	4.48	0.13
	duration of removal	0.35	0.28	1.55	0.25
	presence of pups:removed or resident	−0.86	0.57	2.25	0.15
b) **response variable:** change in socio-positive behaviours (factor 2) compared to the control day					
	intercept	0.86	0.20	–	–
	in the presence of pups	−0.10	0.25	0.42	0.88
	removed or resident female	−0.75	0.25	11.15	0.01
	duration of removal	0.26	0.17	2.27	0.27
	presence of pups:removed or resident	0.22	0.35	0.41	0.55

The increase in socio-negative behaviours in “resident” females in the absence of pups might have to be taken with caution, because it is mainly driven by two outliers. When excluding those two, the confidence interval does no longer cross 0 (mean [95%CI], 0.01 [-0.11–0.14]). Neither the duration of the removal (LMM, *χ*^2^_1_ = 0.1.55, p = 0.250, see Table 2a) nor the presence or absence of pups (LMM, *χ*^2^_1_ = 4.40, p = 0.141, see Table 2a) significantly influenced aggression shown by females.

The change in socio-positive behaviour after a removal in comparison to the previous control day is illustrated in Fig 2B. “Resident” females increased their socio-positive behaviour towards the partner after her return significantly more than “removed” females (*χ*^2^_1_ = 11.15, p = 0.01, see Table 2b). The duration of the removal (4 or 12 hours; *χ*^2^_1_ = 2.30, p = 0.27, see Table 2b) and the presence or absence of pups had no significant effect on the change in behaviours in comparison to the control day (LMM, *χ*^2^_1_ = 0.42, p = 0.88, see Table 2b).

### Maternal care

#### Effect of a separation on the time females spent nursing their young after the reintroduction

**Fig 3:**
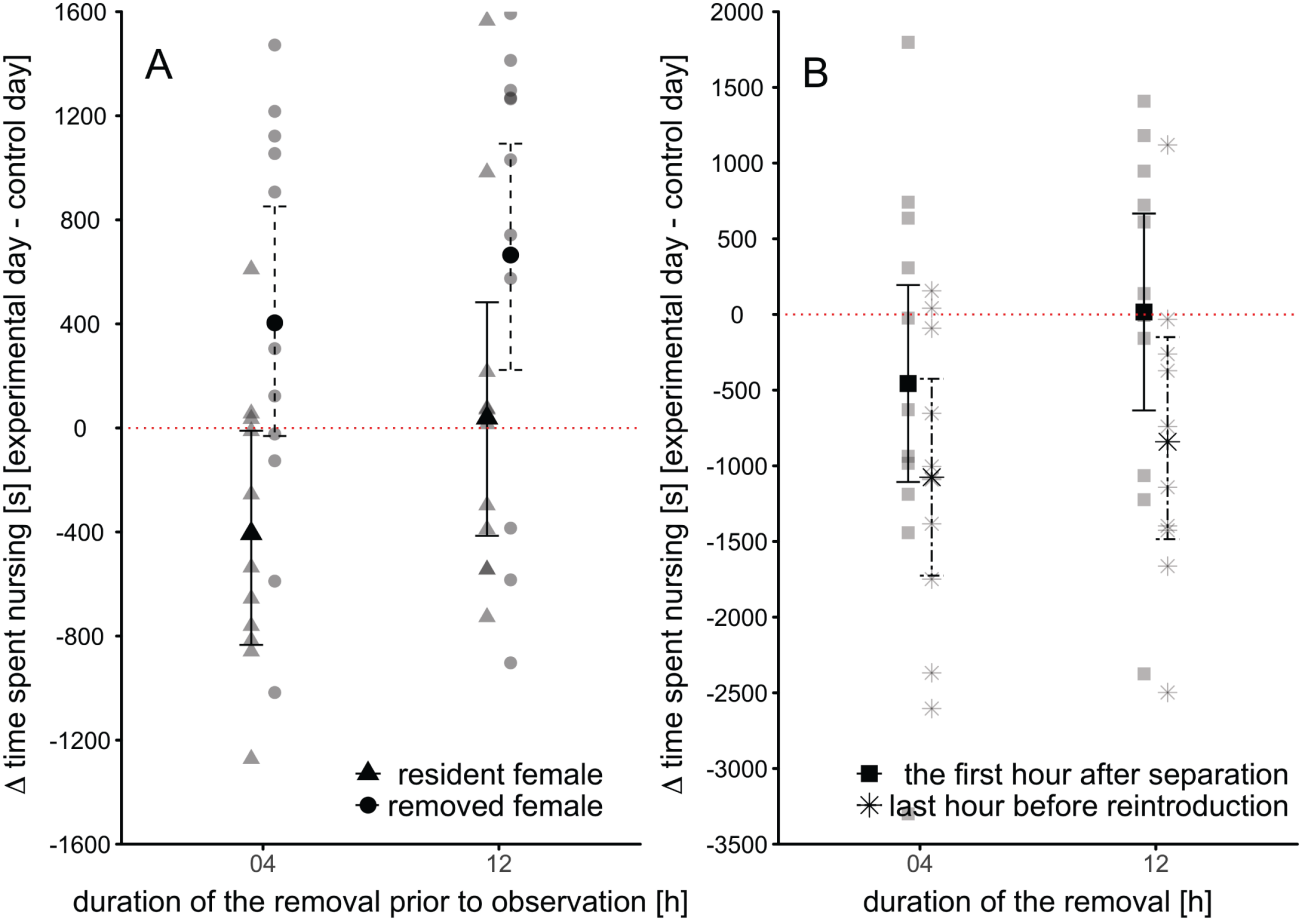
Nursing behaviour. A) The change in time a female spent nursing in comparison to the control day after reintroduction of the “removed” female after 4 or 12 hours. B) The change in time the “resident” female (remaining with the pups) spent nursing young in the absence of her partner. One observation corresponds to the first hour after the separation, the other to the last hour before the reintroduction. Given are model estimates (means) and the 95% CI of the mean. Raw data are plotted in grey.

Females that were removed in the presence of pups tended to increase the amount of time they spent nursing [s] after the reintroduction (mean [95%CI], 404s [-30.72–851.70]). During the same time, “resident” females significantly decreased the time they spent nursing in comparison to the control day (-406.1s [-833.75–-10.80]). Overall, the change in the time spent nursing tended to be more positive in the 12 hours vs. the 4 hours removal (LMM, *χ*^2^_1_ = 6.602, p = 0.053), with “resident” females not significantly decreasing their nursing effort after the 12 hours removal (see Fig 3A).

#### Nursing effort of the resident female during removal

“Resident” females remaining with the pups showed no significant increase in the time spent nursing in the absence of their partner in the first hour after the separation (see Fig 3B). In the last hour before the reintroduction of the “removed” female (see Fig 3B) females significantly decreased their nursing effort compared to the control day. There was, however, no significant difference between the first and the last hour of the observation session (LMM, *χ*^2^_1_ = 4.86, p = 0.160, see Table 3b). The duration of the removal (4 or 12 hours) equally did not significantly affect the time “resident” females spent nursing (LMM, *χ*^2^_1_ = 1.29, p = 0.575, see Table 3b).

**Table 3:** Summary statistics for the linear mixed models. Given are the estimates and SE for the analyses of the change in (a) the time the “resident” and “removed” female spent nursing after the reintroduction compared to the control day and (b) the time the “resident” female spent nursing in the absence of the “removed” female compared to the control day. The intercept in both models corresponds to “resident” female after or during the 4 hour removal.

	explanatory variable	estimate	SE	*χ*^2^	p
a) **response variable:** change in the time females spent nursing after reintroduction [s] compared to the control day					
	intercept	−406.09	213.85	–	–
	removed or resident female	810.27	302.43	6.53	0.07
	duration of removal	444.73	185.78	6.60	0.05
	duration of removal:removed or resident	−184.00	262.74	0.49	0.51
b) **response variable:** change in time the “resident” female spent nursing [s] in the absence of her partner					
	intercept	−1075.18	340.72	–	–
	last or first hour of the separation	618.91	481.85	4.86	0.16
	duration of removal	233.88	493.74	1.29	0.58
	last or first hour:duration of removal	238.85	689.90	0.13	0.73

## Discussion

Our results reveal no evidence for punishment or coercion as a way of enforcing cooperation among communally nursing females. Females did not aggressively punish partners for not providing maternal care during the 4 or 12 hour removal, nor did they compensate for their partners’ absence by increasing nursing effort. Furthermore, we found that “resident” females showed significantly more socio-positive behaviours after the return of a “removed” female both in the presence and absence of pups.

### Why no punishment?

Contrary to what we would expect if punishment served as a mechanism to secure equal investment by females, “resident” females did not punish removed females after their return. Instead we found an increase of socio-positive behaviours (irrespective of whether pups were present or absent). Such a finding raises the question whether punishment is a suitable strategy in this situation. Postpunishment behaviour by the punished individual could take many different forms and only one of them—increasing cooperative behaviour—would be beneficial for the aggressor [44]. Instead of promoting cooperation in communally nursing house mice, aggression by one female towards the other might rather result in avoidance behaviour, maybe up to the point where the harassed female abandons the nest. In that scenario, the punisher would be left to raise the relatively large communal litter on her own. House mice can increase milk production when demand is increased [37, 45] but they are limited in the amount of milk they can produce and might therefore not be able to fully compensate for their partner’s absence [37]. When females are unable to sustain the whole litter, they kill some of the pups [13], which would not be in the interest of any of the females. Being unable to discriminate between own and other offspring further means that females may make mistakes and kill own young. By punishing a “lazy” partner, females would therefore risk to lose the partner, resulting in a partial litter loss and likely higher fitness costs than those associated with bearing a larger share of the maternal investment. We found an increase in socio-positive behaviours after the “removed” female was returned, both in the presence and absence of pups (see Fig 2B), indicating that “resident” females may encourage their partner to stay in the group, as we would expect if the costs of losing a partner are high.

We assumed that aggression leading to an eviction is associated with high costs for a female, making punishment a real threat in this species. Based on observed levels of aggression among unrelated and unfamiliar females (see results) and aggression towards female intruders in house mice [46], we would expect that females face difficulties when trying to integrate into a new group (after having been forced to leave their former group). However, the exact costs for a female associated with having to leave her own group and the effect this might have on her future reproductive success have not been quantified. Further research should focus on assessing both the costs for a female having to leave a group and for a female to stay behind with not only her own, but also the other female’s pups.

We hypothesise therefore that in contrast to cooperative breeding species in which the breeding pair can raise offspring also in the absence of helpers, the costs of losing a partner in communally nursing mice (that proved to be compatible) are too high to allow for the evolution of punishment. Our findings add to a growing list of examples questioning the importance of punishment or coercion in group living animals apart from humans [32, 47–49]. Coercion as a tool to stabilise cooperation in a public good might be more effective in humans, where reputation has been discussed to play role in the evolution of punishment [50].

Conceptually it is difficult to explain how punishment will increase helping behaviour or maternal care. Punishment will result in not performing the punished behaviour again. Associating punishment with a behaviour that was not performed such as increased helping or nursing, on the other hand, is harder to conceive in animals that lack the ability to communicate the intention, as it is possible in humans [32]. The fact that the punished individual can react in various ways, of which most may not be beneficial for the punisher [44], might therefore hinder the evolution of punishment.

There is evidence that elevated stress hormone levels increase pup feeding behaviour in a cooperative breeder (meerkat, *Suricata suricatta*, [51]). Punishment or aggression could therefore promote cooperation by generally increasing stress levels. However, high levels of escalating aggression in the same species (meerkat) were shown to lead to evictions and stress induced abortions, rather indicating that aggression serves as a mechanism to suppress reproduction and not to promote helping behaviour [52]. To our knowledge there is no evidence yet linking an increase in stress induced helping behaviour to punishment.

### Would females benefit from reduced investment?

Alternatively, we could turn the question around and ask what benefits might females gain from lowering investment into a communal nest in the first place that would make punishment necessary. We know from earlier experiments that female house mice invest (milk produced) according to the total number of pups in the nest [15], which provides scope for exploitation. Lowering overall investment into the communal nest when the proportion of own offspring is small would seem beneficial, but was not observed [15]. In this experiment females that were forced to lower their investment through separation from the offspring (“removed” females), were not punished by the “resident” females after their return. If not punishment, what may prevent a female from lowering her investment in such a situation, or in the most extreme case, to abandon the communal nest and leave the litter solely in her partner’s care (intraspecific brood parasitism)? One possible explanation is that physiological constraints prevent females from lowering investment. Milk production in females is determined by the total number of pups suckling [15, 37, 45] and females might therefore have only limited options to modify their lactational effort.

Alternatively, females might not benefit from lowering their investment, or abandoning the communal nest. Reducing investment is only beneficial if the social partner compensates for the loss in investment, otherwise the overall reduction in maternal care may jeopardise offspring growth or survival and create costs that are higher than what the female gains by lowering her investment. Our data show no evidence for immediate compensation in investment by the other female (see Fig 3B), measured as time a female spent nursing. At the end of the removal “resident” females spent significantly less time nursing the young, which could be simply because they emptied their mammary glands. The physiological processes involved in up-regulating milk production might take some time and would likely not be visible within the duration of even 12 hours. This might also explain why the “removed” female increased the time she spent nursing after having been returned to the offspring (see Fig 3A); her separation likely resulted in a build-up of milk in her mammary glands. We cannot exclude that in the longer term females would at least partly compensate for their partner’s absence, as for example in case of the partner’s death. Mice have a postpartum oestrus and in good condition are known to be concurrently pregnant and lactating [53, 54]. Under non-food limited conditions, a female might therefore gain only little time until the birth of the next litter by abandoning her offspring. While she can avoid or lower the burden of lactation, resulting in a shorter inter-birth interval or in a larger next litter, this might not outweigh the cost of losing part of her current litter, especially if the probability to reproduce again is low.

Furthermore, females preferentially communally nurse with relatives [55], which decreases the fitness costs of overinvesting into the joint litter, because females may gain indirect fitness benefits.

### Aggression before the onset of reproduction

While we did not find evidence for punishment, aggression between female house mice was very pronounced before the formation of communal litters. In our study, females were unfamiliar and unrelated at the beginning of the experiment, which resulted in frequent aggression before the onset of reproduction. Aggression reflects competition over reproduction in social groups of females, which is expected to be elevated among unrelated and unfamiliar females [56]. Juvenile familiarity developed among sisters reared together rarely if ever results in such aggressive competition over reproduction [56]. Immigration of previously unfamiliar females in a group nevertheless occurs in house mice [57], which is why we chose unfamiliar and unrelated females, to maximise the conflict potential and therefore the likelihood for punishment. Almost every second pair of females (12 out of 25) had to be separated prematurely to prevent serious injuries, which likely would have resulted in the death of one of the partners. Under natural conditions, we expect one of the females to leave the group in such situations.

Females thus seemed to be very discriminative about what females they tolerated as a member of their group. Such high levels of selectiveness before the onset of breeding could indicate that females have limited options to avoid exploitation after the formation of a communal nest. Females are unable to recognise their own offspring [13], which prevents them from preferentially nursing own pups or from removing them from the communal nest. As a consequence, once they are part of a communal nest, females can only stay and raise the joint litter or abandon their own pups, which might make partner choice beforehand very important. And indeed, there is evidence from both laboratory studies [58] and a wild population [59] that choice plays an important role in communal nursing. The mechanisms used in selecting a partner, however, are still unknown [60].

## Conclusion

To conclude, female house mice i) did not punish “lazy” partners and ii) did not increase the time spent nursing to compensate their partner’s absence, at least not within a time period of 4 or even 12 hours. Punishment thus seems not to serve as a mechanism to stabilise cooperation in this communally breeding species, further questioning the overall importance of punishment in cooperative non-human animal systems [32, 49].

Overall, there are only few empirical examples of punishment as a mechanism to stabilise cooperation in the context of alloparental care, and all were found in cooperatively and not communally breeding species [30, 31, 33, 61]. In addition, it remains still largely unknown what favoured the evolution of punishment in some cooperative breeders, compared to a growing list of species where punishment seems not to play a role [48, 62, 63]. To address the conceptual criticism raised by Raihani et al. [32] it will be important to analyse what aspects of a punished individual’s behaviour will be modified, and how variable those reactions are. Extending that context, Clutton-Brock and Parker [64] had included risk of retaliation in their game theory approach. Punishment may easier evolve in despotic situations, with a pronounced dominance hierarchy, where a punisher does not risk retaliation by the punished individual. In egalitarian situations, instead, other mechanisms as negotiating over the amount of investment during cooperation might be favoured. Ideally, empirical studies will include the fitness consequences of punishment both for the punisher and the punished partner to clarify the conditions under which punishment is stabilized.

## Supporting information

S1 Table Summary of exploratory factor analysis results using maximum likelihood estimation (N = 174). (PDF)

S2 Table Correlation matrix of the six behavioural traits used in the factor analysis (N = 174). (PDF)

S1 File Data used for analyses. (CSV)
