# Supplementary material for: No evidence for punishment in communally nursing female house mice (Mus musculus domesticus)
Source: PLoS One. 2017 Jun 22;12(6):e0179683. doi: 10.1371/journal.pone.0179683 (PMC5480973; doi:10.1371/journal.pone.0179683)
Supplement: S1 Table — (PDF) [file pone.0179683.s001.pdf]

S1 Table Summary of exploratory factor analysis results using Maximum Likelihood Estimation (N = 174)

	Factor loadings	
behaviours	Socio-negative behaviours (1)	Socio-positive behaviours (2)
resting with body contact	-0.05	-0.25
allogrooming	-0.07	**0.33**
chasing	**0.99**	0.05
biting	**0.99**	0.07
sniffing nose	0.09	**0.71**
sniffing anogenital area	**0.46**	**0.63**
