# Supplementary material for: No evidence for punishment in communally nursing female house mice (Mus musculus domesticus)
Source: PLoS One. 2017 Jun 22;12(6):e0179683. doi: 10.1371/journal.pone.0179683 (PMC5480973; doi:10.1371/journal.pone.0179683)
Supplement: S2 Table — (PDF) [file pone.0179683.s002.pdf]

S2 Table Correlation matrix of the six behavioural traits used in the factor analysis (N = 174)

	resting with body contact	sniffing nose	sniffing anogenital area	chasing	biting	allogrooming
resting with body contact	1.0	-0.23	-0.13	-0.06	-0.07	-0.08
sniffing nose	–	1.0	0.49	0.12	0.14	0.21
sniffing anogenital area	–	–	1.0	0.49	0.51	0.20
chasing	–	–	–	1.0	0.99	-0.04
biting	–	–	–	–	1.0	-0.04
allogrooming	–	–	–	–	–	1.0
